# VEXAS Syndrome and IgG4-Related Disease: Coincidental Association or Pathogenetic Link?

**DOI:** 10.31138/mjr.230126.epr

**Published:** 2026-03-01

**Authors:** Senol Kobak, Mehmet Hilmi Dogu, Mehmet Aydogan, Zehra Cagla Karakoc, Binnur Simsek

**Affiliations:** 1 Department of Rheumatology, Istinye University Faculty of Medicine, Istanbul, Türkiye;; 2 Department of Haematology, Istinye University Faculty of Medicine, Istanbul, Türkiye;; 3 Department of Chest Diseases, Liv Hospital, Istanbul, Türkiye;; 4 Department of Infectious Diseases, Istinye University Faculty of Medicine, Istanbul, Türkiye;; 5 Department of Gastroenterology, Istinye University Faculty of Medicine, Istanbul, Türkiye

**Keywords:** VEXAS syndrome, IgG4-related disease, association, pathogenetic link

VEXAS syndrome is a haemato-inflammatory condition caused by somatic mutation of UBA-1 gene which is the major E1 enzyme that initiates ubiquitylation. Ubiquitylation is essential for various cellular processes such as cell cycle progression, DNA damage response, and inflammatory signalling pathways.^1^ Dysregulation of the ubiquitin-proteasome system results in susceptibility to infection, lymphoproliferative disorders, autoinflammatory diseases, and malignancy. VEXAS syndrome often manifests with overlapping rheumatologic and hematologic clinical features. It usually affects middle and older age, predominantly men, but the true prevalence of the syndrome in the population is not known yet. The most common clinical features included recurrent fever, arthralgia/arthritis, pulmonary involvement, skin lesions, vasculitis, and/or thromboembolic events. IgG4-RD is a progressive inflammatory disease characterised by either a diffuse infiltration of inflammatory tissue or solid inflammatory masses that might involve various organs.^2^ The most commonly affected organs are lacrimal and salivary glands, pancreas, retroperitoneum, orbita, and lungs. Diagnosis is made according to clinical, radiological and histopathological findings. Typical histopathological features include storiform fibrosis, dense lymphoplasmacytic infiltrates, and obliterative phlebitis. From a rheumatologist’s perspective, the VEXAS syndrome and IgG4-RD both are newly defined rare diseases that may mimicking known rheumatologic diseases or may coexist with them. Although they have different etiopathogenesis, they may sometimes show similar and overlapping clinical symptoms (**Table 1**). Herein, we report the coexistence of VEXAS syndrome and IgG4-RD.

**Table 1.** VEXAS syndrome vs. IgG4-RD: similarities and differences.

**Features**	**VEXAS syndrome**	**IgG4-RD**
**Age**	Elderly	Elderly
**Sex (predominantly)**	Male	Male
**Clinical findings**		
**Constitutional**	fatigue, weight loss, fever	fatigue, weight loss
**Joint**	asymmetric mono/oligoarthritis	asymmetric mono/oligoarthritis
**Skin**	nodules, papules, and plaques neutrophilic dermatitis	nodules, papules, and plaques lympho-plasmocytic dermatitis
**Eyes**	orbital pseudotumor scleritis/episcleritis/uveitis/retinal vasculitis	orbital pseudotumor dacryoadenitis
**Lung**	neutrophilic alveolitis pleuritis	solid nodular pattern broncho vascular pattern alveolar interstitial pattern round-shaped ground glass opacity
**Kidney**	tubulointerstitial nephritis renal peritubular capillaritis	tubulointerstitial nephritis retroperitoneal fibrosis hydronephrosis
**Vascular**	aortitis, aneurysm, thrombo-emboli, any type of vasculitis	aortitis/periaortitis, aneurysm
**Neurologic**	stroke	stroke
**Heart**	pericarditis, heart failure	pericarditis, cardiac mass
**Exocrine glands**	none	sialadenitis, pancreatitis, cholangitis, thyroiditis
**Lymph nodes**	lymphadenopathy	lymphadenopathy
**Laboratory**	high ESH/CRP high IgG, IgG4, IgE polyclonal gammopathy macrocytic anaemia, cytopenias UBA1 gene mutation	high ESH/CRP high IgG, IgG4, IgE polyclonal gammopathy chronic disease anaemia hypocomplementaemia
**Histopathology**	bone marrow vacuolisation affecting myeloid and erythroid precursor cells	storiform fibrosis, dense lymphoplasmacytic infiltrates obliterative phlebitis
**Mimicker of:**	vasculitis, connective tissue disease, autoinflammatory disease, relapsing polychondritis	sarcoidosis, Sjögren syndrome, primary biliary cirrhosis, primary sclerosing cholangitis
**Treatment**	corticosteroids cDMARDs (MTX, MFM, CyP) Tocilizumab JAKi, Secukinumab Azacytidine IVIG ASCT	corticosteroids cDMARDs (MTX, MFM, CyP) Tocilizumab Rituximab Bortezomib İnfliximab, Abatacept
**Prognosis**	depends on organ involved high risk of malignancy (MDS)	depends on organ involved high risk of malignancy (diffuse large cell lymphoma and pancreatic cancer)

MDS: Myelodysplastic syndrome; ESH: erythrocyte sedimentation rate; CRP: C-reactive protein; cDMARDs: conventional disease modifying anti-rheumatic drugs; IVIG: intravenous immunoglobulin; MTX: methotrexate: MFM: mycophenolate mofetil; CyP: Cyclophosphamide; JAKi: Janus kinase inhibitors; ASCT: allogenic stem cell transplantation.

**Figure 1.**
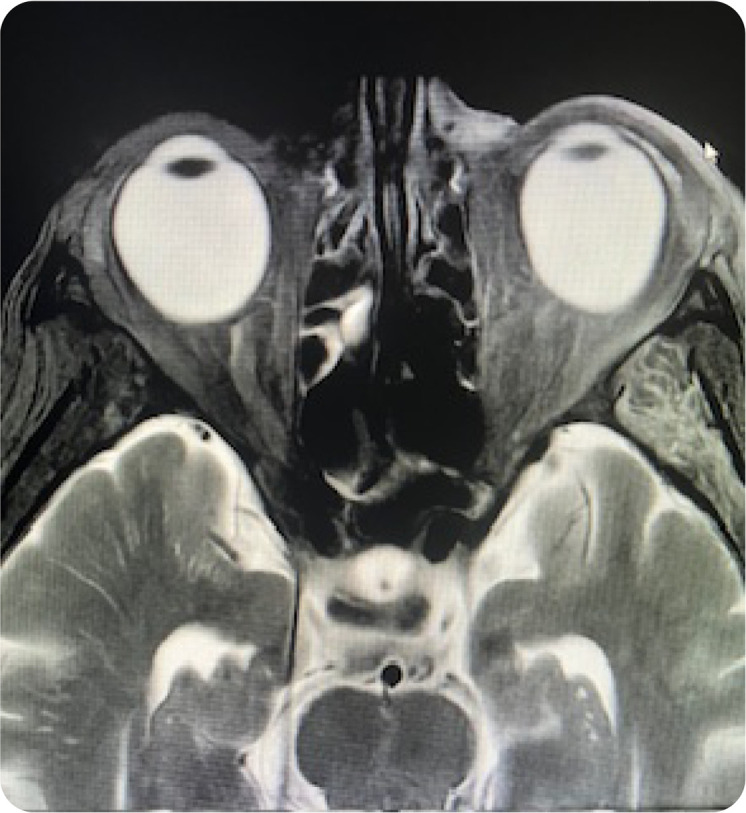
Orbital MRI showing increased signal intensity in the subcutaneous tissues of the left orbital preseptal area, partial retroorbital involvement, and mild proptosis.

Our patient was a 68-year-old male who presented with fatigue, weight loss, skin lesions, dry cough, swelling of the left eyelid, and arthritis. Physical examination revealed arthritis of the left wrist and the 2nd, 3rd, and 4th metacarpophalangeal (MCP) joints, as well as erythema nodosum–like skin lesions on the extremities. Laboratory tests showed elevated inflammatory markers and immunoglobulin levels: C-reactive protein (CRP) 89 mg/L (normal 0–5), erythrocyte sedimentation rate (ESR) 95 mm/h (normal 0–20), IgG 1865 mg/dL (normal 650–1600), IgE 739 IU/mL (normal 0–100), and IgG4 4.25 g/L (normal 0.03–2.01). Antinuclear antibody (ANA), extractable nuclear antigen (ENA) profile, anti-neutrophil cytoplasmic antibody (ANCA), and anti–cyclic citrullinated peptide (anti-CCP) antibody tests were negative. Complete blood count revealed a white blood cell (WBC) count of 6200/μL (normal 4000–9000), haemoglobin (Hb) 11.9 g/dL (normal 13–17), haematocrit (Hct) 37% (normal 40–50), and platelet count 167,000/μL (normal 150,000–400,000). Hypocomplementemia was detected. Thoracic computed tomography (CT) showed peribronchovascular and septal thickening with ground-glass opacities. Bronchoscopy revealed no pathological findings. Positron emission tomography–CT (PET-CT) demonstrated increased fluorodeoxyglucose (FDG) uptake in the subcutaneous tissues of both lower extremities. Skin biopsy from these lesions revealed lymphoplasmacytic dermatitis. Orbital magnetic resonance imaging (MRI) showed an inflammatory process in the left orbital preseptal area, partial retroorbital involvement, and mild proptosis (**Figure 1**). Based on the 2019 ACR/EULAR classification criteria, the patient was diagnosed with IgG4-RD.^3^ Treatment with methylprednisolone 16 mg/day was initiated. After three months, a dramatic clinical and laboratory response was observed. Locomotor system findings, skin lesions, and orbital swelling resolved completely. Laboratory tests showed normalisation of CRP, ESR, and serum IgG and IgG4 levels. Five months later, the patient re-presented with poor general condition, loss of appetite, fever, fatigue, skin lesions, and arthralgia. Laboratory evaluation revealed pancytopenia (WBC 2200/μL, Hb 9.9 g/dL, Hct 30%, platelet count 82,000/μL), macrocytic anaemia (mean corpuscular volume 107 fL), and elevated CRP and ESR. Infectious causes were excluded following consultation with an infectious disease specialist. Given the patient’s advanced age, male sex, fever, skin lesions, pancytopenia, macrocytic anaemia, and musculoskeletal involvement, VEXAS syndrome was suspected. Genetic testing identified a pathogenic *UBA1* (Met41Thr) mutation, consistent with VEXAS syndrome. Bone marrow biopsy performed by a haematologist revealed minimal megakaryocytic dysplasia. The methylprednisolone dose was increased to 32 mg/day. One month later, the patient’s symptoms resolved and laboratory abnormalities normalised. He remains under follow-up in the rheumatology and haematology outpatient clinics.

Here, we report the coexistence of VEXAS syndrome and IgG4-RD in a 68-year-old male patient. Although the typical clinical presentations of IgG4-RD differ from those of VEXAS—such as a more indolent course without fever—there is overlap in their autoinflammatory manifestations, including skin lesions, constitutional and musculoskeletal symptoms, elevated acute-phase reactants, and multiorgan involvement (**Table 1**). The exact mechanisms by which UBA1 mutations lead to inflammation remain unknown. Increased inflammation is driven by mutant myeloid cells that survive despite the somatic mutation.^4^ Patients with VEXAS syndrome exhibit highly activated inflammatory pathways, including tumour necrosis factor, interleukin-6, and interferon-γ. Activation of multiple cytokine cascades results in elevated acute-phase reactant levels, a hall-mark laboratory finding in VEXAS.^5^ Alterations have also been observed in B-lymphocyte repertoires, including atypical B-cell differentiation, loss of immature B cells, and expansion of monocyte populations. Dys-regulated ubiquitination and aberrant T-cell responses may contribute to B-cell activation and increased IgG4 production.^6^ Persistent low-grade inflammation in VEXAS syndrome may sustain elevated IgG4 levels, as chronic inflammatory conditions are often associated with increased IgG4 production. Recently, Saur et al. retrospectively evaluated serum IgG4 levels in nine patients with VEXAS syndrome^7^ and found that 44% had markedly elevated IgG4 levels, with a positive correlation between CRP, ESR, and IgG4 levels. Elevated IgG4 levels have also been reported in various other autoinflammatory and autoimmune diseases.^8^ From a rheumatologist’s perspective, both VEXAS syndrome and IgG4-RD are newly defined rare diseases that may mimic known rheumatologic conditions or coexist with them. Although their etiopathogenesis differs, they may present with similar and overlapping clinical features. Patients previously diagnosed with vasculitis, connective tissue disease, retroperitoneal fibrosis, orbital pseudotumor, or autoinflammatory disease have later been found to have VEXAS syndrome or IgG4-RD in light of recent data.^9^ Notably, patients with VEXAS syndrome or IgG4-RD may also fulfil or partially meet established diagnostic or classification criteria for other clinical conditions.^10^ Despite our patient meeting ACR/EULAR criteria for IgG4-RD, it remains possible that the initial diagnosis represented misclassification rather than true coexistence of both diseases. Rheumatologists should consider VEXAS syndrome in elderly men (typically after the fifth decade of life) presenting with adult-onset autoinflammation, multiorgan involvement, recurrent fever, skin inflammation, asymmetric mono- or oligoarthritis, relapsing polychondritis with pulmonary involvement, vasculitis of any type, and myelodysplastic syndrome–like features in peripheral blood.

In conclusion, we describe a case of VEXAS syndrome coexisting with IgG4-RD, which, to our knowledge, is the first reported in the literature. Although these diseases have distinct etiopathogenesis, they share demographic and clinical similarities. At present, the hypothesis of a shared pathogenetic link remains speculative, and further studies are needed.
